# Identification of Potential Metabolic Markers of Hypertension in Chinese Children

**DOI:** 10.1155/2021/6691734

**Published:** 2021-08-24

**Authors:** Jiahong Sun, Min Zhao, Liu Yang, Xue Liu, Lucia Pacifico, Claudio Chiesa, Bo Xi

**Affiliations:** ^1^Department of Epidemiology, School of Public Health, Cheeloo College of Medicine, Shandong University, Jinan, Shandong, China; ^2^Department of Nutrition and Food Hygiene, School of Public Health, Cheeloo College of Medicine, Shandong University, Jinan, Shandong, China; ^3^Department of Maternal and Child Health, Sapienza University of Rome, Rome, Italy; ^4^Institute of Translational Pharmacology, National Research Council, Rome, Italy

## Abstract

**Background:**

Studies in adults have shown that several metabolites across multiple pathways are strongly associated with hypertension. However, as yet, to our knowledge, no study has investigated such association in childhood. We, therefore, compared the serum metabolite profile of children with normal and elevated blood pressure (BP) to identify potential metabolic markers and pathways that could be useful for the assessment of pediatric hypertension.

**Methods:**

The study included 26 hypertensive children (age range, 6–11 years) and 26 age- and sex-matched ones with normal BP, who were recruited from the baseline survey of the Huantai Childhood Cardiovascular Health Cohort Study. Ultrahigh-performance liquid chromatography-quadrupole time-of-flight-mass spectrometry was performed to assess the serum metabolite profile. Logistic regression analysis was used to select significant metabolites associated with hypertension after adjustment for body mass index, waist circumference, and lipid profile. Kyoto Encyclopedia of Genes and Genomes (KEGG) and MetaboAnalyst were utilized to search for the potential pathways of metabolites.

**Results:**

A total of 45 and 34 metabolites were preliminarily screened in positive and negative modes, respectively (variable importance in the projection (VIP) > 1.0 and *P* < 0.05). After adjustment for the false discovery rate, 7 and 1 differential metabolites in the positive and negative modes, respectively, remained significant (VIP > 1.0 and *q* < 0.05). These metabolites were mainly involved in amino acid metabolism and glycerophospholipid metabolism. Among these, two significant metabolites including ethanolamine and 2-methyl-3-hydroxy-5-formylpyridine-4-carboxylate displayed an area under the curve value of 0.820 (95% confidence interval, 0.688–0.951), with a sensitivity of 0.846 and a specificity of 0.769.

**Conclusion:**

The untargeted metabolomics approach effectively identified the differential serum metabolite profile in children with and without hypertension. Notably, two metabolites including ethanolamine and 2-methyl-3-hydroxy-5-formylpyridine-4-carboxylate exhibited a good discriminative ability to identify children with hypertension, providing new insights into potential mechanisms of pediatric hypertension.

## 1. Introduction

Hypertension ranks as the predominant risk factor for cardiovascular disease (CVD) and related mortality worldwide [1, 2]. In addition to its high prevalence in adults, hypertension has recently shown a rapid rise in prevalence among children and adolescents, trending to manifest at an earlier age. Based on 179,561 children and adolescents aged 3–20 years, a recent meta-analysis showed that the global prevalence of pediatric hypertension, defined as having an elevated blood pressure (BP) at all three different visits, was about 3.0% [3]. Compelling evidence has shown a positive association between pediatric hypertension and target organ damage, such as microalbuminuria, left ventricular hypertrophy, and carotid-intima media thickness [4], all of which are known to predict CVD outcomes in adulthood [5].

Hypertension is a complex disease involving multiple pathways and organs. As such, omic approaches provide the advantage of identifying novel mechanisms of hypertension to help further dissect and characterize the pathophysiology of the disorder [6]. The metabolomics, one of many “omics” quantifying the changes of several metabolites in organisms and representing the end product of exogenous and endogenous determinants [7], might be useful for unraveling the altered metabolomic pathways of hypertension [6]. Recent studies in adults have shown that amino acids, as well as lipid metabolites across multiple pathways, are strongly associated with the development of hypertension [8–11]. For instance, the Bogalusa Heart Study involving 1,249 participants (427 African-Americans, mean age 47 years; 822 whites, mean age 48 years) reported the significant associations of BP phenotypes with 24 novel metabolites, such as amino acid and nucleotide metabolites from histidine, pyrimidine, or tryptophan metabolism subpathways, vitamin and cofactor or xenobiotic metabolites from ascorbate and aldarate metabolism subpathways, and lipid metabolites from eicosanoid and sphingolipid metabolism subpathways [8]. Although associations between metabolites and BP phenotypes have already been observed in the adult population, as yet, to our knowledge, no study has assessed such associations in children. This is a significant issue given the potential influence of age on metabolic profile [12]. Thus, further studies on the relationship between metabolites and BP in the pediatric population might be warranted.

In the present study, using an untargeted profiling approach, we aimed to explore metabolites associated with hypertension (defined as having an elevated BP at all three visits) in children recruited from the baseline survey of the Huantai Childhood Cardiovascular Health Cohort Study.

## 2. Materials and Methods

### 2.1. Study Population and Blood Sample Collection

Participants included in this study were recruited within the baseline survey of the Huantai Childhood Cardiovascular Health Cohort Study. The survey was designed to investigate the impacts of influencing factors on target organ damage in childhood and adolescence and CVD outcomes in adulthood. The baseline survey included 1,505 children who were recruited from one primary school in Huantai County, Zibo City, China, between November 2017 and January 2018. Participants with hypertension (defined as having an elevated BP at all three visits) accounted for 1.9% of the cohort [13].

For the present study, we included a sample of 26 hypertensive children (aged 6–11 years and without a history of antihypertensive medication use) and 26 age- and sex-matched children with normal BP. Complete information on anthropometric variables, blood biochemical parameters, and BP measurements (at all three visits) was available for both cases and controls. Parents or guardians of all participants provided informed written consent, and the study was approved by the research ethics committee of the School of Public Health, Shandong University.

A total of 5 mL overnight-fasting peripheral venous blood was collected. The serum was separated by centrifugation at 3000 rpm for 15 minutes, immediately frozen in liquid nitrogen, and then stored at −80°C until required.

### 2.2. Definition of Hypertension

BP was measured following the standard protocols by trained investigators using validated BP-measuring devices for children (OMRON HEM-7012, Japan) [14]. The last two values of three consecutive BP readings at each visit were used for calculating mean BP values. Elevated BP at each visit was defined as BP ≥ 95^th^ percentile values by age, sex, and height based on the Chinese BP references for children and adolescents [15]. Hypertension was defined as persistently elevated BP at three different clinic visits (with a 2-week interval between two adjacent visits).

### 2.3. Metabolomic Profile

Serum sample preparation for the metabolic profile followed standard protocols that have been described elsewhere [16, 17]. Blank samples (75% MeOH in water) and pooled quality control (QC) samples (pooling of aliquots of all samples) were also detected [18]. In brief, 400 *μ*L of the precooled extraction solvent (V methanol: V acetonitrile = 1 : 1) was added to 100 *μ*L of serum, vortexed (30 s), ultrasound-treated (10 min), and incubated for 1 h at −20°C. The precipitated proteins were centrifuged at 12000 rpm for 15 min at 4°C. The supernatant was transferred to a clean tube, dissolved with a 100 *μ*L water/acetonitrile (1 : 1) solution, then vortexed (30 s), ultrasound-treated (10 min) at 4°C, centrifuged at 12000 rpm for 15 min at 4°C and then separated and subjected to metabolite analysis by ultrahigh-performance liquid chromatography-quadrupole time-of-flight-mass spectrometry (UHPLC-QTOF/MS) using a UHPLC system (1290 series, Agilent Technologies, USA) coupled to a QTOF mass spectrometer (Agilent 6550 iFunnel QTOF, Agilent Technologies, USA). The mobile phase consisted of 25 mM NH_4_OH and 25 mM NH_4_Ac in water (pH = 9.75). Tandem mass spectrometry (MS) data acquisition was performed using another QTOF mass spectrometer (TripleTOF6600, SCIEX, Canada).

### 2.4. Data Preprocessing and Annotation

MS raw data (.wiff) files were converted to the mzXML format using ProteoWizard and processed by *R* package XCMS (version 3.6). After preprocessing steps including filtering noisy data and baseline calibration, peak alignment, deconvolution, recognition, alignment of retention time, and imputation of missing data, a data matrix consisting of the retention time, mass-to-charge ratio (m/*z*) values, and peak intensity was generated. *R* package CAMERA was used for peak annotation. Metabolic features detected in less than 50% of QC samples as well as the metabolic features with a relative standard deviation above 30% were discarded. An in-house MS2 database was applied for metabolite identification, and a metabolic reaction network- (MRN-) based recursive algorithm (MetDNA) [19] was used to expand metabolite annotations without the need for a comprehensive standard spectral library.

### 2.5. Statistical Analysis

Continuous variables were presented as median (P25–P75), while categorical variables as *n* (%). The nonparametric test and chi-square test were used to compare continuous and categorical variables, respectively, between the hypertensive and normotensive groups.

Principal component analysis (PCA) was first used to detect grouping trends and outliers. Orthogonal partial least-squares discriminant analysis (OPLS-DA) was used to understand global metabolic changes between hypertensive and normotensive groups and the variable importance in the projection (VIP) scores. *R*^2^ scores indicate model performance, and Q^2^ scores estimate reproducibility based on cross validation. The permutation test was performed to check the predictive robustness and ability. The intercept value of *Q*^2^ < 0 and the left *R*^2^ and Q^2^ values lower than the right original points indicate the good fit, reliability, and robustness of the models. Potential metabolic biomarkers were selected based on VIP values more than 1.00 and a false discovery rate adjusted *P* value (i.e., *q* value) less than 0.05. Logistic regression analysis was used to examine the association between 1-log-transformed metabolites and hypertension. A basic model (model 1) was adjusted for body mass index. A second model (model 2) was adjusted for body mass index plus waist circumference. Model 3 was adjusted for variables entered in model 2 plus lipid profile including triglycerides, low-density lipoprotein cholesterol, and high-density lipoprotein cholesterol. Potential pathways analyses were performed using the Kyoto Encyclopedia of Genes and Genomes (KEGG) and MetaboAnalyst. *P* < 0.05 indicates a statistically significant difference.

To evaluate the classification performance, the area under the curve (AUC) value of receiver operating characteristic (ROC) was used. Statistical analyses were performed using the *R* platform (version 3.6) and SAS (version 9.4), except for OPLS-DA using SIMCA 14.0 (Umetrics AB, Umea, Sweden).

## 3. Results

### 3.1. Characteristics of Participants

Table 1 presents the clinical characteristics of 26 children with hypertension and 26 ones with normal BP (median age, 9.56 years; 53.8% boys). Compared to children with normal BP, those with hypertension had significantly higher body mass index (22.01 vs. 16.01 kg/m^2^), waist circumference (75.53 vs. 57.83 cm), systolic BP (122.00 vs. 104.00 mmHg), diastolic BP (74.50 vs. 59.75 mmHg), low-density lipoprotein cholesterol (2.38 vs. 2.03 mmol/L), triglyceride (0.78 vs. 0.65 mmol/L), and alanine aminotransferase levels (15.50 vs. 12.00 mmol/L) and significantly lower levels of high-density lipoprotein cholesterol (1.48 vs. 1.66 mmol/L).

### 3.2. Metabolomic Analysis

In both positive and negative ionization modes, PCA analysis showed that the tightly clustered QC samples mirrored the stability of UHPLC-QTOF/MS analysis. The OPLS-DA plot indicated the differences in metabolites between the hypertensive and normotensive groups as detected by the positive (Figure S1(a)) and negative (Figure S1(b)) modes. In addition, the permutation plots for the OPLS-DA model showed that *R*^2^ and Q^2^ met the performance of discrimination between the two study groups, highlighting the reliability and accuracy of the statistical model (Figures S1(c) and S1(d)).

### 3.3. Identification of Potential Biomarkers

**Figure 1:**
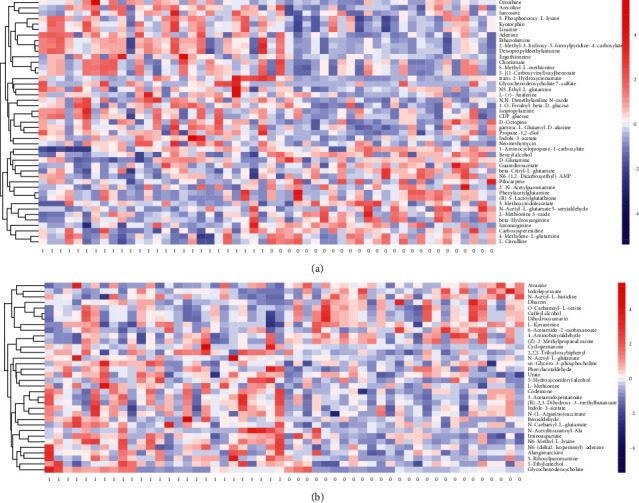
Heatmap of metabolites between hypertensive and normotensive children as detected by the (a) positive mode (1: hypertension; 0: normal blood pressure) and (b) negative mode.

The OPLS-DA model provided significant differences in biomarkers between hypertensive and normotensive groups (Table S1). A total of 45 and 34 metabolites were preliminarily screened in the positive and negative modes, respectively (VIP > 1 and *P* < 0.05). After adjustment for the false discovery rate, 7 and 1 differential metabolites remained significant in the positive and negative modes, respectively (VIP > 1 and *q* < 0.05). The heatmaps of significant biomarkers between hypertensive and normotensive groups as detected in the positive and negative modes are depicted in Figures 1(a) and 1(b), respectively.

### 3.4. Analysis of Metabolic Pathways

**Figure 2:**
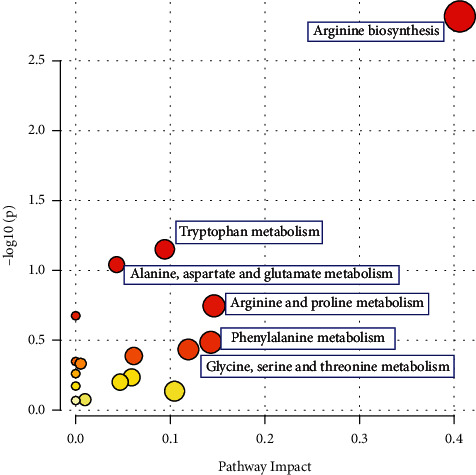
Pathway analysis for hypertensive children vs. normotensive children in the (a) positive mode and (b) negative mode.

Pathway enrichment analyses of the differential metabolites with VIP > 1 and *P* < 0.05 were performed to unravel pathways associated with hypertension in children. As shown in Figure 2 and Table S2, only the arginine biosynthesis pathway was statistically significant (*P* < 0.05) in the positive mode with a high impact power of 0.406. Other pathways, including tryptophan metabolism (impact 0.094), alanine, aspartate, and glutamate metabolism (impact 0.043), arginine and proline metabolism (impact 0.146), phenylalanine metabolism (impact 0.143), glycine, serine, and threonine metabolism (impact 0.119), glycerophospholipid metabolism (impact 0.061), and purine metabolism (impact 0.005), failed to attain statistical significance. Nonetheless, they had impacts greater than 0, suggesting their potential contributions as hypertension-related metabolic pathways.

### 3.5. AUC Analysis for Further Selected Metabolites

**Figure 3:**
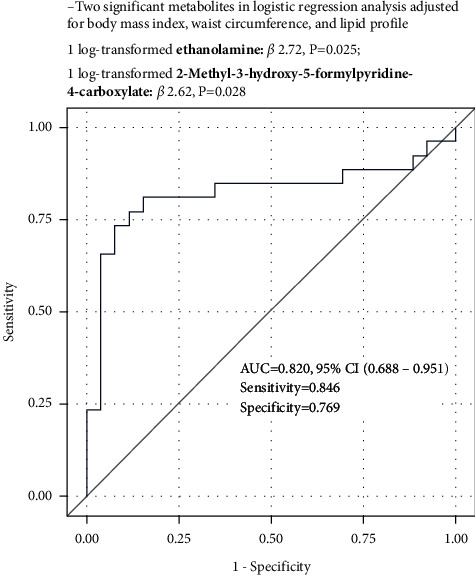
ROC analysis of two metabolites, including ethanolamine and 2-methyl-3-hydroxy-5- formylpyridine-4-carboxylate, for identification of children with hypertension.

In model 1, three metabolites (ethanolamine (*β* = 1.55, *P*=0.032), kyotorphin (*β* = 5.35, *P*=0.049), and 2-methyl-3-hydroxy-5-formylpyridine-4-carboxylate (*β* = 1.50, *P*=0.040)) were found to be significantly associated with hypertension with the aid of criteria (i.e., VIP > 1 and the adjusted *q* value < 0.05). In model 2, similar results were obtained. However, in model 3, only ethanolamine (*β* = 2.72, *P*=0.025) and 2-methyl-3-hydroxy-5-formylpyridine-4-carboxylate (*β* = 2.62, *P*=0.028) remained significant (Table 2). Using a logistic regression model, we also performed ROC analysis to calculate the AUC value of two significant metabolites including ethanolamine and 2-methyl-3-hydroxy-5-formylpyridine-4-carboxylate. As shown in Figure 3, the two metabolites showed a good discriminative ability to identify children with hypertension (AUC value: 0.820 (95% confidence interval, 0.688–0.951), sensitivity: 0.846, and specificity: 0.769).

## 4. Discussion

In this study, differential biomarkers between hypertension and normal BP in children were evaluated. A total of 45 and 34 metabolites were preliminarily screened in the positive and negative modes, respectively. The relevant metabolites were determined to be mainly involved in amino acid metabolism and glycerophospholipid metabolism pathways. The two significant metabolites including ethanolamine and 2-methyl-3-hydroxy-5-formylpyridine-4-carboxylate displayed a good discriminative ability to identify hypertension in Chinese children.

### 4.1. Amino Acid Metabolism

Previous studies in adults have found that metabolites associated with BP or hypertension are involved in the amino acid metabolism pathway [6], which represents an important pathway for endothelial cell metabolism and vasculature formation [20]. Alterations in amino acid metabolism have been reported to be associated with CVD through the regulation of vascular homeostasis and immune cell function [21].

We identified many relevant differential metabolites involved in the amino acid pathways, such as N-(L-arginino) succinate, citrulline, and ornithine. It is worth emphasizing that arginine and succinate have been suggested to play important roles in maintaining rhythm homeostasis in spontaneously hypertensive rats [22]. Also, citrulline and ornithine have been recognized as markers of endothelial nitric oxide synthesis capacity, contributing to the regulation of BP [23–25]. Evidence-based reviews from adult populations have shown that oral administration of citrulline, a potential substrate for eNOS, may effectively reduce BP by increasing NO production [26]. While increased arginase activity has been reported in a variety of disease conditions characterized by vascular dysfunction, citrulline and ornithine as arginase inhibitors have been found to alleviate hypertension in diabetic animals [25]. Taken together, these findings suggest that citrulline and ornithine might have beneficial effects on cardiovascular health in adults as well as in children.

### 4.2. Glycerophospholipid Metabolism and Purine Metabolism

Findings from our study show that metabolites involved in the glycerophospholipid metabolism pathway may also play a relevant role in pediatric hypertension. Glycerophospholipid metabolism pathway comprising two different routes, one toward phosphatidylethanolamine and the other toward phosphatidylcholine, has been shown to be a potential metabolic footprint of generation of reactive oxygen species in endothelial cells [27]. The disturbance of this pathway can trigger endothelial cell dysfunction and the development of atherosclerotic lesions [27]. In previous studies involving adult populations, phosphatidylethanolamine has been reported as a marker for hypertension, chronic kidney disease, and CVD [28, 29]. Metabolomic data from the Husermet project have shown that certain metabolic biomarkers involved in glycerophospholipid metabolism are hypertension-related, being implicated in vascular remodeling [10], even in the development of ischemic hypertensive stroke [30]. Oxidized phospholipids may induce endothelial dysfunction by targeting amino acid metabolism [31]. Under conditions of oxidative stress, glycerophospholipids can be transferred to oxidized phospholipids, which may significantly contribute to inflammation in diseased vessels by inducing the expression of adhesion molecules and proinflammatory cytokines on vascular endothelial cells as well as promoting monocyte adhesion and by acting directly on leukocytes. Furthermore, oxidized phospholipids may stimulate reactive oxygen species production, attenuate endothelial-dependent vasorelaxation, induce phenotypic modulation and migration of smooth muscle cells, and stimulate vessel calcification, therefore leading to the development of CVD [32]. Findings from serum metabolomics research on spontaneously hypertensive rats have highlighted the important role played by certain biomarkers involved in glycerophospholipid metabolism on nitric oxide production as well as on vascular smooth muscle cell apoptosis and proliferation [33].

In our pediatric study, ethanolamine and sn-glycero-3-phosphocholine have been found as the most relevant hypertension-related biomarkers among those involved in glycerophospholipid metabolism. Ethanolamine and sn-glycero-3-phosphocholine necessary for the biosynthesis of phosphatidylethanolamine and phosphatidylcholine (KEGG map00564) have been implicated in the regulation of endothelial cell function and in the development of atherosclerotic lesions [27]. In addition, anandamide, which is formed enzymatically by the condensation of arachidonic acid with ethanolamine, has been demonstrated to be relevant in the regulation of the cardiovascular system given its ability to induce vasorelaxation in the vessel [34, 35]. Ethanolamine also affects left ventricle diastolic function and cardiac fibrosis [36]. sn-Glycero-3-phosphocholine has been shown to contribute to vascular dilatation and arterial BP reduction by blocking alpha-adrenergic receptors on a smooth muscle cell line [37]. It is also considered as an inflammatory mediator in endothelial cells implicated in atherosclerosis [38], which is associated with clinical hypertension [39].

Our findings also show that adenine, involved in the purine metabolism pathway, is an important component of nicotinamide adenine dinucleotide phosphate, well known for its role in driving the reactive oxygen species and therefore in promoting inflammation, vascular remodeling, and endothelial dysfunction [40]. Finally, it should be stressed that along with adenine, urate is also one of the differential metabolites involved in purine metabolism, with a potential role in hypertension. It has been demonstrated that among Mexican girls aged 8–14 years, serum urate represented a key metabolite that marked the relationship between intake of sugar-sweetened beverages and higher systolic and diastolic BP [41]. In this respect, of great interest is the recent recommendation to maintain urate levels within the normal range in young (pre-) hypertensive individuals or normotensives with a family history of hypertension, metabolic disorders, or obesity [42].

### 4.3. Clinical Implications

From our findings, in addition to ethanolamine, 2-methyl-3-hydroxy-5-formylpyridine-4-carboxylate displayed a good discriminative ability to identify children with hypertension. The association between 2-methyl-3-hydroxy-5-formylpyridine-4-carboxylate and hypertension has not been investigated previously. Nonetheless, 2-methyl-3-hydroxy-5-formylpyridine-4-carboxylate as an intermediate metabolite in vitamin B6 production has been shown to prevent insulin resistance and vascular dysfunction by regulating cellular homocysteine concentration in the transsulfuration pathway and acetylcholine-induced endothelium-dependent relaxation [43, 44]. Further studies with larger sample sizes are needed to confirm the ability of these metabolites to identify children with hypertension.

### 4.4. Strengths and Limitations

To the best of our knowledge, by using an untargeted metabolomics platform, we first explored in children the relationship of the metabolite profile with hypertension and found two significant metabolites with a good discriminative ability to identify children with hypertension. In addition, hypertensive children in this study were strictly defined as having an elevated BP at all three visits, thus avoiding false-positive cases. However, several limitations should be acknowledged. First, although two metabolites (ethanolamine and 2-methyl-3-hydroxy-5-formylpyridine-4-carboxylate) had a good discriminative ability to identify children with hypertension, a causal relationship could not be inferred because of the case-control study design. Second, our sample size was limited to 26 cases with hypertension and 26 controls with normal BP. Because of its small sample size, our study should be considered exploratory until validated in future large cohort studies. However, the small sample size can still provide statistical confidence for our results because of substantial changes in metabolites, which could be easily measured [16]. Third, participant recruitment from China involved one single center. Fourth, the limited number of potential metabolites (i.e., ethanolamine and 2-methyl-3-hydroxy-5-formylpyridine-4-carboxylate) impedes us to further perform analysis using the machine learning method. Fifth, only metabolomics of pediatric hypertension was addressed. Future studies with a multiomics approach, including genomes and epigenetics, are needed to gain a comprehensive and global picture of mechanisms involved in pediatric hypertension.

## 5. Conclusion

In this study including Chinese children, most differential metabolites associated with hypertension were those involved in amino acid metabolism and glycerophospholipid metabolism pathways. Notably, ethanolamine and 2-methyl-3-hydroxy-5-formylpyridine-4-carboxylate exhibited a good discriminative ability to identify hypertension among study participants.

## Figures and Tables

**Table 1:** Characteristics of study participants.

	Overall (*N* = 52)	Control (*N* = 26)	Hypertension (*N* = 26)	*P* value
BMI, kg/m^2^	17.38 (15.35, 22.05)	16.01 (14.92, 16.98)	22.01 (19.58, 24.85)	<0.001
WC, cm	60.38 (56.06, 75.69)	57.83 (54.83, 60.04)	75.53 (66.60, 80.50)	<0.001
SBP, mmHg	109.25 (104.00, 122.00)	104.00 (98.38, 106.13)	122.00 (115.88, 127,13)	<0.001
DBP,mmHg	68.25 (59.13, 74.50)	59.75 (57.88, 62.5)	74.50 (70.00, 77.63)	<0.001
GLU, mmol/L	4.78 (4.43, 5.08)	4.60 (4.28, 5.05)	4.82 (4.64, 5.12)	0.087
HDL-C, mmol/L	1.57 (1.30, 1.84)	1.66 (1.39, 2.01)	1.48 (1.20, 1.65)	0.038
LDL-C, mmol/L	2.24 (1.93, 2.52)	2.03 (1.79, 2.49)	2.38 (2.11, 2.73)	0.019
TG, mmol/L	0.72 (0.53, 0.91)	0.65 (0.48, 0.76)	0.78 (0.67, 1.16)	0.002
CHOL, mmol/L	4.07 (3.65, 4.56)	4.00 (3.62, 4.37)	4.13 (3.89, 4.66)	0.341
CREA, mmol/L	42.65 (37.08, 47.40)	40.45 (35.18, 46.83)	43.65 (38.25, 49.05)	0.426
ALT, mmol/L	14.00 (12.00, 16.00)	12.00 (11.00, 16.00)	15.50 (12.75, 18.00)	0.020
AST, mmol/L	23.00 (21.00, 25.00)	23.00 (22.00, 25.00)	22.00 (20.00, 24.25)	0.124

Data were presented as median (P25–P75) or n (%). BMI, body mass index; WC, waist circumference; SBP, systolic blood pressure; DBP, diastolic blood pressure; GLU, glucose; HDL-C, high-density lipoprotein cholesterol; LDL-C, low-density lipoprotein cholesterol; TG, triglycerides; CHOL, cholesterol; CREA, creatinine; ALT, alanine aminotransferase; AST, aspartate transaminase.

**Table 2:** Selected metabolites (VIP > 1 and adjusted *q* value < 0.05) according to logistic regression analysis.

	Model 1		Model 2		Model 3	
	*β*	*P* value	*β*	*P* value	*β*	*P* value
N6-Methyl-L-lysine	1.90	0.34c4	1.77	0.390	2.42	0.315
Benzyl alcohol	−2.50	0.102	−2.48	0.113	−2.41	0.161
L-Citrulline	−1.53	0.533	−1.55	0.527	−2.40	0.372
Linatine	5.64	0.065	6.00	0.061	5.66	0.104
Adenine	2.69	0.157	2.71	0.157	2.65	0.205
Ethanolamine	1.55	0.032	1.56	0.033	2.72	0.025
2-Methyl-3-hydroxy-5-formylpyridine-4-carboxylate	1.50	0.040	1.52	0.041	2.62	0.028
Kyotorphin	5.35	0.049	5.53	0.048	6.04	0.071

Model 1: adjusted for body mass index; Model 2: adjusted for body mass index and waist circumference; Model 3: adjusted for body mass index, waist circumference, and lipid profile (triglycerides, low-density lipoprotein cholesterol, and high-density lipoprotein cholesterol).

## Data Availability

Data are available from the corresponding author upon request (Bo Xi, email: xibo2007@126.com).

## Supplementary Materials

Supplementary Materials Figure S1: OPLS-DA plots and permutation plots in positive and negative modes ((a) the OPLS-DA plot in the positive mode; (b) the OPLS-DA plot in the negative mode; (c) the permutation plot in the positive mode; (d) the permutation plot in the negative mode). Table S1: differential serum metabolites between hypertension and normal blood pressure in children. Table S2: pathway analysis of differential metabolites.

## References

[1] Causes of Death Collaborators G. B. D. (2018). Global, regional, and national age-sex-specific mortality for 282 causes of death in 195 countries and territories, 1980–2017: a systematic analysis for the global burden of disease study 2017. *Lancet*.

[2] Yusuf S., Joseph P., Rangarajan S. (2020). Modifiable risk factors, cardiovascular disease, and mortality in 155 722 individuals from 21 high-income, middle-income, and low-income countries (PURE): a prospective cohort study. *The Lancet*.

[3] Sun J., Steffen L. M., Ma C., Liang Y., Xi B. (2017). Definition of pediatric hypertension: are blood pressure measurements on three separate occasions necessary?. *Hypertension Research*.

[4] Rao G. (2016). Diagnosis, Epidemiology, and management of hypertension in children. *Pediatrics*.

[5] Yang L., Magnussen C. G., Yang L., Bovet P., Xi B. (2020). Elevated blood pressure in childhood or adolescence and cardiovascular outcomes in adulthood. *Hypertension*.

[6] Arnett D. K., Claas S. A. (2018). Omics of blood pressure and hypertension. *Circulation Research*.

[7] Nikolic S. B., Sharman J. E., Adams M. J., Edwards L. M. (2014). Metabolomics in hypertension. *Journal of Hypertension*.

[8] He W. J., Li C., Mi X. (2020). An untargeted metabolomics study of blood pressure: findings from the bogalusa heart study. *Journal of Hypertension*.

[9] Zhao H., Liu Y., Li Z. (2018). Identification of essential hypertension biomarkers in human urine by non-targeted metabolomics based on UPLC-Q-TOF/MS. *Clinica Chimica Acta*.

[10] Ke C., Zhu X., Zhang Y., Shen Y. (2018). Metabolomic characterization of hypertension and dyslipidemia. *Metabolomics*.

[11] Dietrich S., Floegel A., Weikert C. (2016). Identification of serum metabolites associated with incident hypertension in the European prospective investigation into cancer and nutrition-potsdam study. *Hypertension*.

[12] Saito K., Maekawa K., Kinchen J. M., Tanaka R., Kumagai Y., Saito Y. (2016). Gender- and age-associated differences in serum metabolite profiles among Japanese populations. *Biological & Pharmaceutical Bulletin*.

[13] Yang L., Ma C. W., Zhao M. (2020). Detection of hypertension based on measurements at three occasions in different days and its relationship with obesity in children. *Chinese Journal of Epidemiology*.

[14] Meng L. L., Hou D. Q., Shan X. Y. (2013). Accuracy evaluation of Omron HEM-7012 electronic sphygmomanometers in measuring blood pressure of children and adolescents. *Chinese Journal of Hypertension*.

[15] Fan H., Yan Y. K., Mi J. (2017). Updating blood pressure references for Chinese children aged 3–17 years. *Chinese Journal of Hypertension*.

[16] Dunn W. B., Broadhurst D., Begley P. (2011). Procedures for large-scale metabolic profiling of serum and plasma using gas chromatography and liquid chromatography coupled to mass spectrometry. *Nature Protocols*.

[17] Wang J., Zhang T., Shen X. (2016). Serum metabolomics for early diagnosis of esophageal squamous cell carcinoma by UHPLC-QTOF/MS. *Metabolomics*.

[18] Smith C. A., Want E. J., O’Maille G., Abagyan R., Siuzdak G. (2006). XCMS: processing mass spectrometry data for metabolite profiling using nonlinear peak alignment, matching, and identification. *Analytical Chemistry*.

[19] Shen X., Wang R., Xiong X. (2019). Metabolic reaction network-based recursive metabolite annotation for untargeted metabolomics. *Nature Communications*.

[20] Li X., Kumar A., Carmeliet P. (2019). Metabolic pathways fueling the endothelial cell drive. *Annual Review of Physiology*.

[21] Nitz K., Lacy M., Atzler D. (2019). Amino acids and their metabolism in atherosclerosis. *Arteriosclerosis, Thrombosis, and Vascular Biology*.

[22] Wang H., Wang X., Qi D. (2020). Establishment of the circadian metabolic phenotype strategy in spontaneously hypertensive rats: a dynamic metabolomics study. *Journal of Translational Medicine*.

[23] Wood K. C., Cortese-Krott M. M., Kovacic J. C. (2013). Circulating blood endothelial nitric oxide synthase contributes to the regulation of systemic blood pressure and nitrite homeostasis. *Arteriosclerosis, Thrombosis, and Vascular Biology*.

[24] Fike C. D., Summar M., Aschner J. L. (2014). L-citrulline provides a novel strategy for treating chronic pulmonary hypertension in newborn infants. *Acta Paediatrica*.

[25] El-Bassossy H. M., El-Fawal R., Fahmy A. (2012). Arginase inhibition alleviates hypertension associated with diabetes: effect on endothelial dependent relaxation and NO production. *Vascular Pharmacology*.

[26] Khalaf D., Krüger M., Wehland M., Infanger M., Grimm D. (2019). The effects of oral l-arginine and l-citrulline supplementation on blood pressure. *Nutrients*.

[27] Xu W., Qian M., Huang C. (2019). Comparison of mechanisms of endothelial cell protections between high-density lipoprotein and apolipoprotein A-I mimetic peptide. *Frontiers in Pharmacology*.

[28] Zheng Y., Li Y., Rimm E. B. (2016). Dietary phosphatidylcholine and risk of all-cause and cardiovascular-specific mortality among US women and men. *American Journal of Clinical Nutrition*.

[29] Taesuwan S., Vermeylen F., Caudill M. A., Cassano P. A. (2019). Relation of choline intake with blood pressure in the national health and nutrition examination survey 2007–2010. *American Journal of Clinical Nutrition*.

[30] Guo X., Li Z., Zhou Y. (2019). Metabolic profile for prediction of ischemic stroke in Chinese hypertensive population. *Journal of Stroke and Cerebrovascular Diseases*.

[31] Hitzel J., Lee E., Zhang Y. (2018). Oxidized phospholipids regulate amino acid metabolism through MTHFD2 to facilitate nucleotide release in endothelial cells. *Nature Communications*.

[32] Philippova M., Resink T., Erne P., Bochkov V (2014). Oxidised phospholipids as biomarkers in human disease. *Swiss Medical Weekly*.

[33] Jiang H., Shen Z., Chu Y. (2015). Serum metabolomics research of the anti-hypertensive effects of Tengfu Jiangya tablet on spontaneously hypertensive rats. *Journal of Chromatography B*.

[34] Carnevale L. N., Arango A. S., Arnold W. R., Tajkhorshid E., Das A. (2018). Endocannabinoid virodhamine is an endogenous inhibitor of human cardiovascular CYP2J2 epoxygenase. *Biochemistry*.

[35] Movahed P., Evilevitch V., Andersson T. L. G. (2005). Vascular effects of anandamide and N -acylvanillylamines in the human forearm and skin microcirculation. *British Journal of Pharmacology*.

[36] Yamamoto K., Takahashi Y., Mano T. (2004). N-methylethanolamine attenuates cardiac fibrosis and improves diastolic function: inhibition of phospholipase D as a possible mechanism. *European Heart Journal*.

[37] Smith K. A., Cornett L. E., Norris J. S., Byers L. W., Muirhead E. E. (1982). Blockade of alpha-adrenergic receptors by analogues of phosphatidylcholine. *Life Sciences*.

[38] Nishimukai M., Maeba R., Ikuta A. (2014). Serum choline plasmalogens-those with oleic acid in sn− 2-are biomarkers for coronary artery disease. *Clinica Chimica Acta*.

[39] Safar M. E. (2018). Arterial stiffness as a risk factor for clinical hypertension. *Nature Reviews Cardiology*.

[40] Montezano A. C., Dulak-Lis M., Tsiropoulou S., Harvey A., Briones A. M., Touyz R. M. (2015). Oxidative stress and human hypertension: vascular mechanisms, biomarkers, and novel therapies. *Canadian Journal of Cardiology*.

[41] Perng W., Tang L., Song P. X. K. (2019). Urate and nonanoate mark the relationship between sugar-sweetened beverage intake and blood pressure in adolescent girls: a metabolomics analysis in the element cohort. *Metabolites*.

[42] De Becker B., Borghi C., Burnier M., van de Borne P. (2019). Uric acid and hypertension. *Journal of Hypertension*.

[43] Gu X., Al Dubayee M., Alshahrani A. (2020). Distinctive metabolomics patterns associated with insulin resistance and type 2 diabetes mellitus. *Frontiers in molecular biosciences*.

[44] Liu Z., Li P., Zhao Z. H. (2016). Vitamin B6 prevents endothelial dysfunction, insulin resistance, and hepatic lipid accumulation in apoe (−/−) mice fed with high-fat diet. *Journal of diabetes research*.

