# Supplementary material for: Identification of Potential Metabolic Markers of Hypertension in Chinese Children
Source: Int J Hypertens. 2021 Aug 24;2021:6691734. doi: 10.1155/2021/6691734 (PMC8410451; doi:10.1155/2021/6691734)
Supplement: Supplementary Materials — Figure S1: OPLS-DA plots and permutation plots in positive and negative modes ((a) the OPLS-DA plot in the positive mode; (b) the OPLS-DA plot in the negative mode; (c) the permutation plot in the positive mode; (d) the permutation plot in the negative mode). Table S1: differential serum metabolites between hypertension and normal blood pressure in children. Table S2: pathway analysis of differential metabolites. [file 6691734.f1.zip › 6691734.f1/Supplementary Description_6691734.docx]

Supplementary Description:

**Figure S1.** OPLS-DA plots and permutation plots in positive and negative modes (**A**, OPLS-DA plot in positive mode; **B**, OPLS-DA plot in negative mode; **C**, permutation plot in positive mode; **D**, permutation plot in negative mode)

Table S1 Differential serum metabolites between hypertension and normal blood pressure in children

Table S2 Pathway analysis of differential metabolites
