# Supplementary figures and images for: Identification of Potential Metabolic Markers of Hypertension in Chinese Children
Source: Int J Hypertens. 2021 Aug 24;2021:6691734. doi: 10.1155/2021/6691734 (PMC8410451; doi:10.1155/2021/6691734)

A

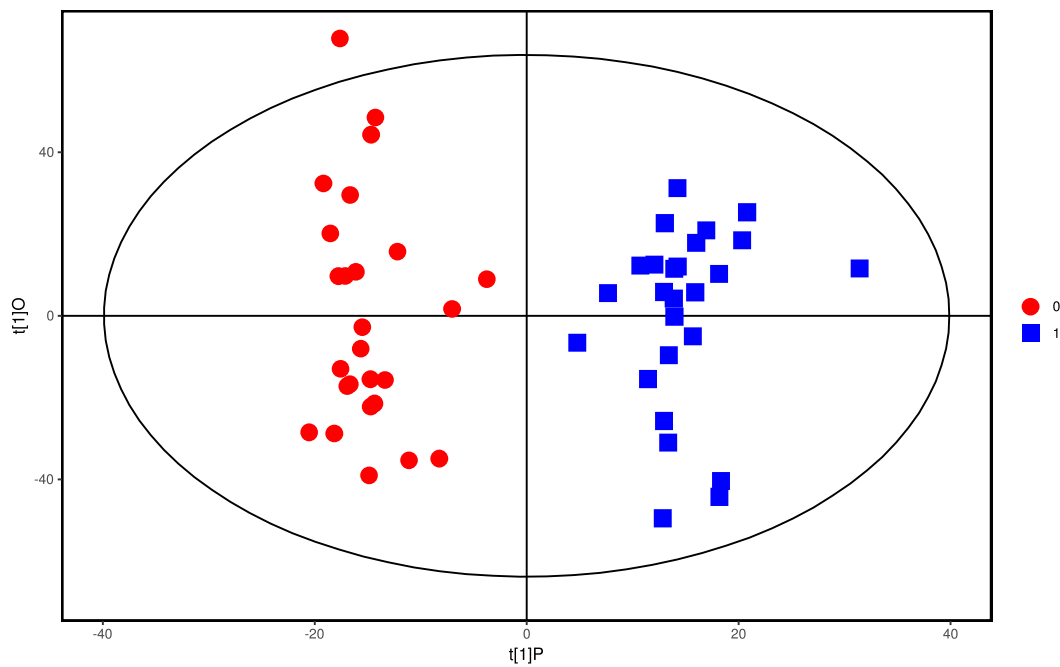

B

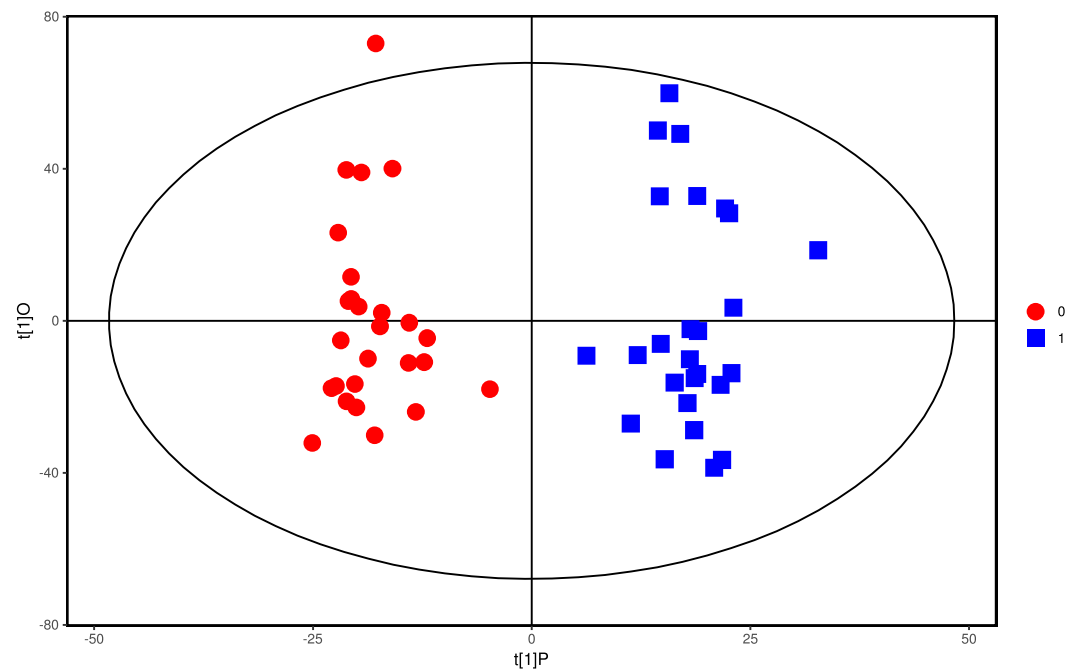

C

Intercepts:  $R^2Y(\text{cum}) = (0, 0.89)$ ,  $Q^2(\text{cum}) = (0, -0.86)$ 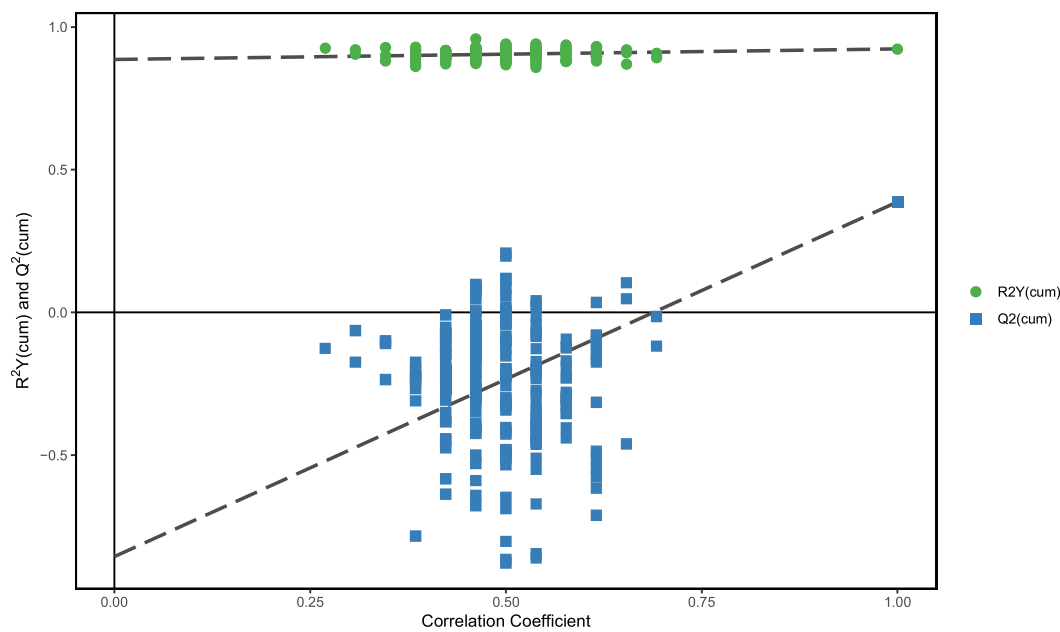

D

Intercepts:  $R^2Y(\text{cum}) = (0, 0.9)$ ,  $Q^2(\text{cum}) = (0, -0.56)$ 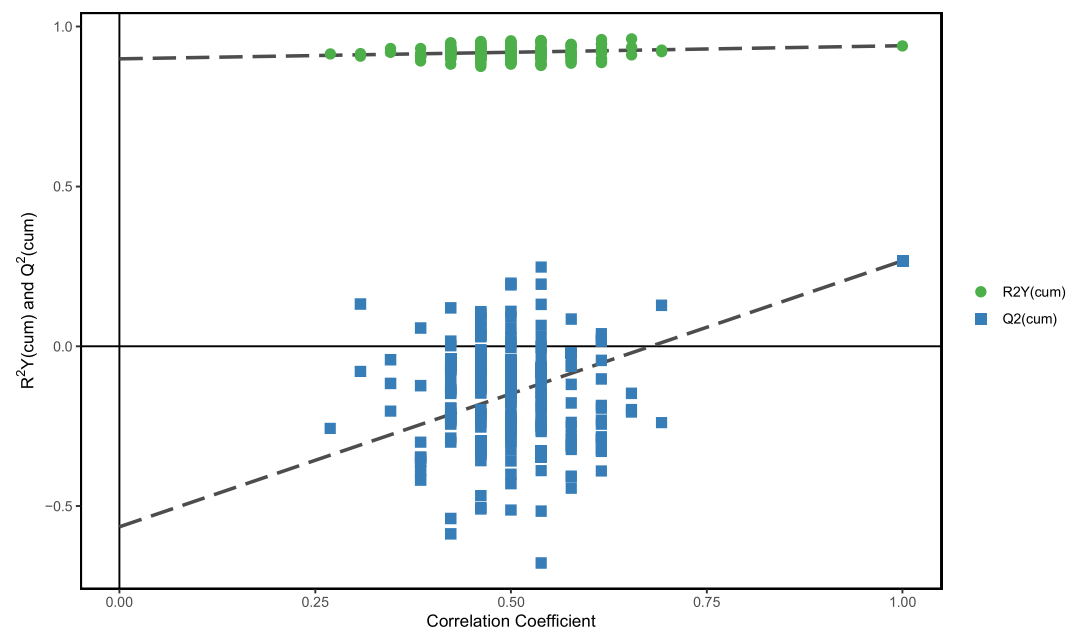

Figure S1

Supplement: Supplementary Materials — Figure S1: OPLS-DA plots and permutation plots in positive and negative modes ((a) the OPLS-DA plot in the positive mode; (b) the OPLS-DA plot in the negative mode; (c) the permutation plot in the positive mode; (d) the permutation plot in the negative mode). Table S1: differential serum metabolites between hypertension and normal blood pressure in children. Table S2: pathway analysis of differential metabolites. [file 6691734.f1.zip › 6691734.f1/Figure S1 (1).pdf]
