# Supplementary material for: Identification of Potential Metabolic Markers of Hypertension in Chinese Children
Source: Int J Hypertens. 2021 Aug 24;2021:6691734. doi: 10.1155/2021/6691734 (PMC8410451; doi:10.1155/2021/6691734)
Supplement: Supplementary Materials — Figure S1: OPLS-DA plots and permutation plots in positive and negative modes ((a) the OPLS-DA plot in the positive mode; (b) the OPLS-DA plot in the negative mode; (c) the permutation plot in the positive mode; (d) the permutation plot in the negative mode). Table S1: differential serum metabolites between hypertension and normal blood pressure in children. Table S2: pathway analysis of differential metabolites. [file 6691734.f1.zip › 6691734.f1/TableS1-2_2021.7.27.doc]

Supplementary Table 1 Differential serum metabolites between hypertension and normal blood pressure in children

| **Metabolites** | **VIP** | ***P*-value** | ***q*-value** | **Fold Change** | **Log_Foldchange** |
| --- | --- | --- | --- | --- | --- |
| **Positive mode** |  |  |  |  |  |
| Phenylacetylglutamine | 2.292512191 | 0.031083233 | 0.288983749 | 0.582727487 | -0.779106732 |
| Guanidinoacetate | 1.986287272 | 0.045032243 | 0.333604833 | 0.815174411 | -0.29481933 |
| Ergothioneine | 1.353143648 | 0.034464167 | 0.299984564 | 1.507844581 | 0.592487732 |
| Arecoline | 2.54877046 | 0.003642156 | 0.109945932 | 1.344789238 | 0.427380085 |
| trans-2-Hydroxycinnamate | 1.863677107 | 0.018842057 | 0.237013408 | 1.432958804 | 0.518997134 |
| Iminoarginine | 1.998802578 | 0.047016653 | 0.339488096 | 0.903308519 | -0.14670928 |
| D-Glutamine | 2.141492435 | 0.02445456 | 0.262502757 | 0.931535941 | -0.102316663 |
| 4-Methylene-L-glutamine | 2.059197177 | 0.012633452 | 0.200315369 | 0.875324571 | -0.192110027 |
| Glycochenodeoxycholate 7-sulfate | 1.654217647 | 0.040663158 | 0.319833256 | 2.476889589 | 1.30852956 |
| 1-Aminocyclopropane-1-carboxylate | 2.189159704 | 0.011502749 | 0.191990457 | 0.868872624 | -0.2027834 |
| L-Citrulline | 2.814716235 | 0.000328291 | 0.041931915 | 0.808408866 | -0.30684295 |
| S-Methyl-L-methionine | 1.999755823 | 0.0370446 | 0.307480502 | 1.399632993 | 0.485048578 |
| D-Octopine | 1.556562718 | 0.047750871 | 0.341590069 | 1.157883204 | 0.211489736 |
| gamma-L-Glutamyl-D-alanine | 2.63696029 | 0.002143803 | 0.08278527 | 1.249268934 | 0.321084084 |
| L-Methionine S-oxide | 2.346176403 | 0.008374068 | 0.166267258 | 0.684451828 | -0.546979087 |
| Benzyl alcohol | 2.576439641 | 0.000128884 | 0.029309994 | 0.71677342 | -0.480410956 |
| Indole-3-acetate | 1.918909698 | 0.008295394 | 0.165588605 | 1.593089787 | 0.67182758 |
| N,N-Dimethylaniline N-oxide | 2.073440751 | 0.000803347 | 0.055737133 | 1.960832389 | 0.97146622 |
| 2-Methyl-3-hydroxy-5-formylpyridine-4-carboxylate^*^ | 1.595145051 | 0.000368673 | 0.043482013 | 1.594676133 | 0.673263453 |
| beta-Hydroxyarginine | 1.187487398 | 0.032088354 | 0.292407612 | 0.648003676 | -0.625926098 |
| Chorismate | 2.272421457 | 0.040682427 | 0.319896359 | 1.608723998 | 0.68591683 |
| N5-Ethyl-L-glutamine | 1.122192842 | 0.046049243 | 0.336657651 | 1.227306686 | 0.295495803 |
| Deisopropyldeethylatrazine | 1.680062505 | 0.000655191 | 0.052875997 | 1.571474136 | 0.652118528 |
| N-Acetyl-L-glutamate 5-semialdehyde | 1.754673014 | 0.029687009 | 0.283990881 | 0.747994561 | -0.418900315 |
| Ornithine | 1.65758892 | 0.013585677 | 0.206706314 | 1.739402044 | 0.798591434 |
| Neomethymycin | 2.315522321 | 0.00466145 | 0.125139232 | 1.471616437 | 0.557401696 |
| (R)-S-Lactoylglutathione | 2.239012111 | 0.024536425 | 0.262866032 | 0.522289265 | -0.937079044 |
| beta-Citryl-L-glutamate | 2.666064542 | 0.000756741 | 0.054927638 | 0.887824767 | -0.17165314 |
| Sarcosine | 2.040119267 | 0.015117888 | 0.216527683 | 1.329424556 | 0.410801907 |
| Propane-1,2-diol | 1.794241698 | 0.007464313 | 0.15796608 | 1.286240592 | 0.363160525 |
| L-(+)-Anaferine | 1.649435135 | 0.047380044 | 0.340533336 | 1.305247178 | 0.384323039 |
| 1-O-Feruloyl-beta-D-glucose | 2.196786569 | 0.00509535 | 0.131116107 | 1.706722443 | 0.771228458 |
| 3-[(1-Carboxyvinyl)oxy]benzoate | 1.873705728 | 0.027684356 | 0.276303035 | 1.532455375 | 0.615845064 |
| Isopropylamine | 1.855319131 | 0.009821964 | 0.177917184 | 1.564518027 | 0.645718282 |
| 5-Phosphonooxy-L-lysine | 2.532378086 | 0.002553068 | 0.091209231 | 1.200151701 | 0.263216776 |
| 2'-N-Acetylparomamine | 1.65014499 | 0.043935382 | 0.330219867 | 0.852295512 | -0.230574359 |
| N6-(1,2-Dicarboxyethyl)-AMP | 2.266939588 | 0.009184926 | 0.17286192 | 0.891004089 | -0.166496043 |
| Pilocarpine | 1.32514889 | 0.033732851 | 0.297725599 | 0.806271947 | -0.310661568 |
| Adenine | 2.798375908 | 0.000478327 | 0.04794193 | 1.256693361 | 0.329632668 |
| Carboxyspermidine | 2.123261641 | 0.027571294 | 0.275849103 | 0.848321371 | -0.237317188 |
| Ethanolamine^*^ | 1.692084249 | 0.000209506 | 0.035536409 | 1.608032777 | 0.685296814 |
| CDP-glucose | 1.97034326 | 0.017756494 | 0.231365689 | 1.231065468 | 0.299907487 |
| Kyotorphin | 3.013197911 | 0.000326228 | 0.041845769 | 1.273023238 | 0.348258754 |
| 5-Methoxyindoleacetate | 1.330334515 | 0.028951318 | 0.281242006 | 0.686534127 | -0.542596657 |
| Linatine | 2.955000355 | 0.000449698 | 0.046909578 | 1.215714814 | 0.281804837 |
| **Negative mode** |  |  |  |  |  |
| Cyclopentanone | 2.115856954 | 0.019109164 | 0.127414019 | 2.662804079 | 1.412946283 |
| Caffeyl alcohol | 1.548756269 | 0.025514809 | 0.147406473 | 0.911092342 | -0.134330812 |
| Iminoaspartate | 1.801348128 | 0.037191793 | 0.175401244 | 1.144831601 | 0.195135401 |
| O-Carbamoyl-L-serine | 1.598257864 | 0.027817162 | 0.153862922 | 0.925993816 | -0.110925536 |
| Benzaldehyde | 1.596025513 | 0.031381238 | 0.162840011 | 1.424938225 | 0.510899376 |
| 5-Hydroxyconiferyl alcohol | 1.912029214 | 0.006613947 | 0.080170572 | 1.625101527 | 0.700529852 |
| Dihydrocoumarin | 1.746394315 | 0.012154584 | 0.102616383 | 0.904745181 | -0.144416577 |
| L-Kynurenine | 1.633419865 | 0.016127414 | 0.117069362 | 0.880633166 | -0.183386915 |
| N-Acetylmuramoyl-Ala | 1.719964114 | 0.043571082 | 0.188453104 | 2.3422026 | 1.227865874 |
| Indolepyruvate | 1.991033496 | 0.008425044 | 0.087745365 | 0.604916479 | -0.725192132 |
| Phenylacetaldehyde | 1.758214994 | 0.031604286 | 0.163365037 | 1.389352426 | 0.474412603 |
| N6-Methyl-L-lysine | 3.051196025 | 8.43704E-05 | 0.019090339 | 1.416343778 | 0.502171482 |
| 4-Aminobutyraldehyde | 1.566615637 | 0.02453087 | 0.144530266 | 0.868470583 | -0.203451112 |
| N-Acetyl-L-glutamate | 1.470495793 | 0.048312153 | 0.197671849 | 1.346956126 | 0.429702859 |
| N6-(delta2-Isopentenyl)-adenine | 1.657639015 | 0.003355501 | 0.065143025 | 1.429940273 | 0.515954889 |
| 5-Acetamidopentanoate | 1.604423614 | 0.007952857 | 0.085908178 | 1.355877604 | 0.439226952 |
| Codeinone | 1.797715072 | 0.003064108 | 0.063840479 | 1.301081138 | 0.379710935 |
| Atrazine | 2.029659787 | 0.044979206 | 0.19129492 | 0.73838768 | -0.437549613 |
| Urate | 1.456482408 | 0.036246176 | 0.173472889 | 1.04821068 | 0.067928713 |
| sn-Glycero-3-phosphocholine | 1.335807781 | 0.031263034 | 0.162560124 | 1.141396204 | 0.19079967 |
| (Z)-2-Methylpropanal oxime | 1.608425996 | 0.021157724 | 0.134205835 | 0.886298991 | -0.174134625 |
| L-Methionine | 1.741322254 | 0.002590061 | 0.061258914 | 1.375326445 | 0.459774095 |
| 3-Ethylcatechol | 1.03680214 | 0.041500448 | 0.184270321 | 1.228760571 | 0.297203828 |
| Alangimarckine | 1.358181271 | 0.037638096 | 0.176370777 | 1.340835789 | 0.423132562 |
| Glycochenodeoxycholate | 1.02893342 | 0.041977997 | 0.185189848 | 1.584585398 | 0.664105413 |
| 6-Acetamido-2-oxohexanoate | 1.580362213 | 0.033045216 | 0.166661584 | 0.720748929 | -0.472431306 |
| Indole-3-acetate | 1.484970578 | 0.024947614 | 0.145746641 | 1.18224194 | 0.241525306 |
| Dhurrin | 1.299383234 | 0.039663555 | 0.180619356 | 0.767979146 | -0.380860959 |
| N-Acetyl-L-histidine | 1.689015432 | 0.037801537 | 0.176722736 | 0.817446924 | -0.290803034 |
| N-Carbamyl-L-glutamate | 2.126055849 | 0.005499925 | 0.074788407 | 1.239775485 | 0.310078882 |
| (R)-2,3-Dihydroxy-3-methylbutanoate | 1.421165208 | 0.012216616 | 0.102823668 | 1.407736152 | 0.493376959 |
| N-(L-Arginino)succinate | 1.58508843 | 0.031520923 | 0.163169286 | 1.191346778 | 0.252593415 |
| 2,2',3-Trihydroxybiphenyl | 1.272627829 | 0.001741507 | 0.054394825 | 1.495727084 | 0.58084696 |
| 5-Ribosylparomamine | 1.732292331 | 0.00338691 | 0.065272892 | 2.59294122 | 1.374589501 |

“^*^” indicates metabolites used for ROC analysis.

Supplementary Table 2 Pathway analysis of differential metabolites

| Pathway | Number of differential metabolites/total | Raw *P* | *P_FDR* | Impact | Name of differential metabolites [KEGG ID] |
| --- | --- | --- | --- | --- | --- |
| Arginine biosynthesis | 4/14 | 0.002 | 0.128 | 0.406 | N-(L-Arginino)succinate [C03406]; L-Citrulline [C00327]; L-Ornithine [C00077]; N-Acetyl-L-glutamate [C00624] |
| Tryptophan metabolism | 4/41 | 0.071 | 1 | 0.094 | L-Kynurenine [C00328]; Indolepyruvate [C00331]; 5-Methoxyindoleacetate [C05660]; Indole-3-acetate [C00954] |
| Alanine, aspartate and glutamate metabolism | 3/28 | 0.091 | 1 | 0.043 | N-(L-Arginino)succinate [C03406]; N6-(1,2-Dicarboxyethyl)-AMP [C03794]; beta-Citryl-L-glutamate [C20775] |
| Arginine and proline metabolism | 3/38 | 0.179 | 1 | 0.146 | N(omega)-Hydroxyarginine [C05933]; Guanidinoacetate [C00581]; L-Ornithine [C00077] |
| D-Glutamine and D-glutamate metabolism | 1/6 | 0.211 | 1 | 0.000 | D-Glutamine [C00819] |
| Phenylalanine metabolism | 1/10 | 0.327 | 1 | 0.143 | Phenylacetaldehyde [C00601] |
| Glycine, serine and threonine metabolism | 2/33 | 0.368 | 1 | 0.119 | Guanidinoacetate [C00581]; Sarcosine [C00213] |
| Glycerophospholipid metabolism | 2/36 | 0.410 | 1 | 0.061 | Ethanolamine [C00189]; sn-Glycero-3-phosphocholine [C00670] |
| Nicotinate and nicotinamide metabolism | 1/15 | 0.448 | 1 | 0.000 | Iminoaspartate [C05840] |
| Purine metabolism | 3/65 | 0.466 | 1 | 0.005 | N6-(1,2-Dicarboxyethyl)-AMP [C03794]; Adenine [C00147]; Urate [C00366] |
